# Interaction Mortality: Senescence May Have Evolved because It Increases Lifespan

**DOI:** 10.1371/journal.pone.0109638

**Published:** 2014-10-09

**Authors:** Maarten J. Wensink, Tomasz F. Wrycza, Annette Baudisch

**Affiliations:** 1 Max Planck Research Group 'Modeling the Evolution of Aging, Max Planck Institute for Demographic Research, Rostock, Germany; 2 Leyden Academy on Vitality and Ageing, Leiden, The Netherlands; 3 Max-Planck Odense Center on the Biodemography of Aging, University of Southern Denmark, Odense M, Denmark; University of North Carolina, Greensboro, United States of America

## Abstract

Given an extrinsic challenge, an organism may die or not depending on how the threat interacts with the organism's physiological state. To date, such interaction mortality has been only a minor factor in theoretical modeling of senescence. We describe a model of interaction mortality that does not involve specific functions, making only modest assumptions. Our model distinguishes explicitly between the physiological state of an organism and potential extrinsic, age-independent threats. The resulting mortality may change with age, depending on whether the organism's state changes with age. We find that depending on the physiological constraints, any outcome, be it ‘no senescence’ or ‘high rate of senescence’, can be found in any environment; that the highest optimal rate of senescence emerges for an intermediate physiological constraint, i.e. intermediate strength of trade-off; and that the optimal rate of senescence as a function of the environment is driven by the way the environment changes the effect of the organism's state on mortality. We conclude that knowledge about the environment, physiology and their interaction is necessary before reasonable predictions about the evolution of senescence can be made.

## Introduction

The effect of extrinsically imposed mortality on the evolution of senescence has received considerable attention [1]–[6]. Initially, it was advocated that extrinsically imposed mortality should accelerate, and even cause senescence [1]–[4], an idea that survives to date [7]. This view has been refuted by rigorous modeling [8], [9]. In general, it holds that mortality does not affect evolution if it affects all organisms equally [8]–[10]. The intuitive reason for this is that evolution favors a phenotype (strategy) if it is better at propagation than other strategies. If all strategies are affected equally, no strategy improves relative to others, and selection gradients remain unchanged.

Mortality that does not distinguish between individuals is often called ‘extrinsic mortality’ and modeled as an age-independent parameter in the mortality function of age-structured models [8], [9], [11]–[13]. In these models, extrinsic mortality is a discounting factor in the survival function that cannot be molded in any way by the (fictitious) organism that is studied. However, whether environmental threats result in mortality depends on the interaction of those threats with an organism's physiological state [11], [14], [15]. By adjusting its state, an organism can influence death from environmental causes. In this respect, we highlight that age-independence does not imply state-independence: the relevant state parameters might just happen not to change over age. Indeed, the level of an age-independent term in the mortality function can be molded by the organism's state, and this molding is subject to natural selection.

To investigate mortality-environment interactions from a theoretical perspective, we model a trade-off between an age-independent and an age-dependent mortality term. As an example of a biological rationale for such a model, Wensink et al. [16] have suggested that it could be beneficial from an evolutionary standpoint to attain a state that is unmaintainable by its very nature, causing mortality to be low at young ages, but to increase over time. As a result, death can be postponed to later ages, depending on the magnitude of initial reduction relative to the ensuing increase in mortality with age.

Many of the theoretical models of senescence that have been proposed depend on particular functions [13], [17]–[19]. This suffices for a proof of principle, but leaves one wondering what the result would have been had a different function been used. We postulate general mathematical conditions that characterize the trade-off. As a result, the model does not predict exact patterns of mortality, but rather charts the range of outcomes that can be obtained with specific models that fulfill the formal conditions.

We find that depending on the physiological constraints, any outcome is possible in any environment, be it ‘no senescence’ or ‘high rate of senescence’; that the highest optimal rate of senescence emerges for an intermediate physiological constraint; and that the optimal rate of senescence as a function of the environment is driven by the way the environment changes the effect of the organism's state on mortality. We conclude that predicting the outcome requires knowledge about the interaction of the environment and the organismal physiology: separately, these have little predictive power. We propose, perhaps paradoxically, that senescence may have evolved because it extends lifespan.

### Analysis

Consider the mortality function
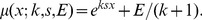
(1)Variable *x* denotes age. Separated from the variable by a semi-colon are the age-independent parameters *k*, *s* and *E*. *E* models the environment, higher values indicating a more challenging environment, *k* is a trade-off parameter that reduces death through the term *E*/(*k*+1) but gives rise to an increase of mortality over age through the term *e^ksx^*, modified by *s*, which models the ‘severity’ of the trade-off (high *s* leads to fast increase in mortality with age for any specified *k*>0). For *k* = 0 mortality is initially higher than for *k*>0, but does not rise further with age, while any increase in *k* reduces mortality initially, but leads to a faster age-related increase in mortality, depending on *s*.

Because there is no strong theoretical basis on which to assume mortality function (1), we define a set of general formal conditions for the mortality function that describes the trade-off. We refer to Appendix S1 for the complete formal description of the model, but the general idea is straightforward. The model has two additive components, *A*(*x*; *k*, *s*) and *B*(*k*). Component *A* depends on age, while component *B* does not. Responsible for the trade-off is parameter *k*. It reduces component *B*, but increases the rate at which *A* increases with age, depending on yet another parameter, *s*. Thus, *k* reduces mortality initially, but gives rise to an age related increase of mortality. The steepness of this increase depends on *s*. Parameters *k* and *s* and variable *x* act in a multiplicative manner, i.e. *ksx*, so that if the organism deteriorates 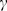 times as fast (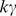), or if deterioration impacts mortality 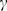 times as much (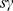), or if 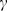 times as much time has passed (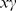), this all has the same effect. For the analysis of the effect of the environment, we consider that component *B* co-depends on a parameter *E*, which models the environmental challenge: *B*(*k*, *E*).

To find *k* ^*^, the optimal *k*, we maximize Darwinian fitness subject to the constraints as formalized. Fitness is given by *r* as the unique real root of the Euler-Lotka equation [20]–[23]:
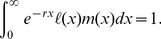
(2)Survival is denoted by 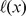; the reproductive rate is denoted by *m*(*x*); and *r* is the intrinsic rate of increase, or the unique real root of equation 2. Survival and the mortality rate are related through
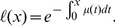
(3)The derivative of *r* with respect to *k* is [19], [24]: 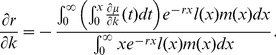
(4)This equation is used to evaluate whether *r* can increase by an increase in *k* under specified circumstances.

For discussions on how to measure the rate of senescence, see [25], [26]. However measured, the rate of senescence will be zero if *k* ^*^ *s* = 0. We use only this property to obtain our results, hence not relying on a particular measure.

## Results

**Figure 1:**
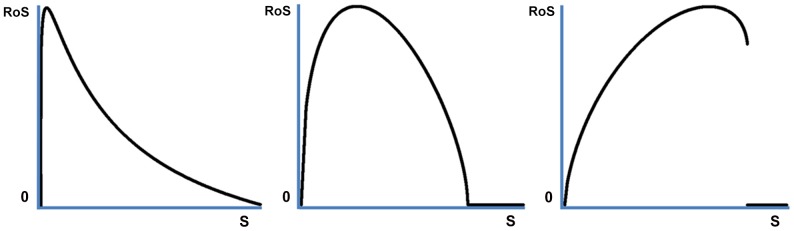
The optimal rate of senescence as a function of *s* for a variety of functions. The rate of senescence ‘RoS’ calculated as *k* ^*^ *s* is given as a function of *s* for a fixed value of *E* given three specific trade-off functions. These graphs demonstrate that a variety of patterns may exist, that may have discontinuities and/or points at which the function is not differentiable. Yet these graphs all have in common that the optimal rate of senescence is zero for *s* = 0, then increases, and then returns to zero for large values of *s* (Appendix S1).

Given our model assumptions, the highest optimal rate of senescence occurs for an intermediate value of *s*, i.e. an intermediate severity of the trade-off. No senescence occurs if *k* ^*^ or *s* equals zero. Hence it follows that the optimal rate of senescence is zero if *s* is zero. It can also be proven that the optimal rate of senescence is greater than zero for at least one *s*>0. In addition, the optimal rate of senescence is zero if *s* is large. This, then, charts the general pattern, proven in Appendix S1: the highest rate of senescence is found for an intermediate physiological constraint *s*. In Figure 1 we present simulations for several specific functions, varying parameter *s*. The optimal rate of senescence starts at zero, increases, and then drops back to zero for large enough *s*. Figure 1 illustrates that there exists a variety of exact patterns, depending on function specifics, that all conform to the general pattern proven in Appendix S1. Whether the optimal rate of senescence is a continuous function of *s* or not, the location of the peak, and other specifics do depend on the exact trade-off equation and on the age-pattern of reproduction, *m*(*x*).

A formal proof of this result is given in Appendix S1, but can be broadly understood from the way *k* and *s* interact. A value of *k* = 0 makes *r* insensitive to *s*, whereas values of *k*>0 imply that larger *s* reduces fitness without bound. Smaller values of *s* imply a slower increase in mortality over age for a specified *k*>0, so that, if *s* is small enough, the initial reduction in mortality outweighs the costs of mortality increasing with age for some *k*>0.

**Figure 2:**
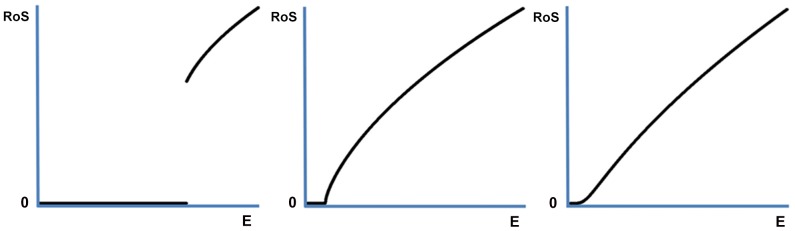
The optimal rate of senescence as a function of *E* for a variety of functions. The rate of senescence ‘RoS’ calculated as *k* ^*^ *s* is given as a function of *E* for a fixed value of *s* given three specific trade-off functions. All these functions have in common that a harsher environment allows for a more favorable perturbation of mortality. This, however, may not be the case in general; these simulations are not a general result (Appendix S1). Notice how discontinuities can be introduced by changing the function specifics.

The optimal rate of senescence as a function of the environment is less straightforward (Appendix S1). If 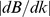 is a monotonously increasing function of the environment, as in equation (1), a harsher environment allows for a larger perturbation of mortality. In this case, a harsher environment would work pro-senescence. If, on the other hand, 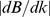 is not a function of the environment, or a decreasing function of the environment, such an effect is not expected. In Figure 2 we present simulations for several specific functions, for all of which 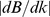 is a monotonously increasing function of *E*. These simulations, while demonstrating a distinct possibility, do not follow from the model as a general result. Whether they apply or not depends on how exactly the environment interacts with organismal physiology.

## Discussion

There are many ways to model the impact of environmental threats on mortality as a function of an organism's state. We have focused on a term that is a function of *ksx* versus an age-independent term, because age-independent mortality by default does not effect evolution [8]–[10]. We show that if an age-independent term of the mortality function is a function of an organism's state, this term can nevertheless be related to senescence, by allowing a trade-off between the mortality rate at age zero versus the rate at which mortality increases with age. Yet, other models may be equally valid representations for environment-state interactions. However, we argue that our model is at least *reasonable*: the interactions may play out the way we have outlined. Rejecting our results for specific circumstances, then, requires the knowledge that the interactions are *not* the way we have modeled them: it requires knowledge of environment-state interactions. Therefore it holds in general that no predictions can be made without knowledge of these interactions.

We found that if 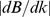 is a monotonously increasing function of *E*, a harsher environment can be pro-senescence, contrary to elementary evolutionary theory, that states that mortality does not affect evolution if it affects all organisms equally [8]–[10]. Alongside other plausible explanations such as density effects [8], this finding could explain why a harsher environment was positively correlated with senescence in particular studies, e.g. [27].

Life history models are as general as their assumptions are minimal. The choice of a parametric function for the purpose of theoretical modeling is itself an assumption. It limits a model's predictive power. For instance, if mortality in a life history optimization model is captured by the (parametric) Gompertz function [28], [29], one is left wondering what the result would be if mortality were captured by a different function. Detailed prediction of a mortality trajectory requires a deep understanding of its underlying determinants. In life history models, it is rarely the case that exact mortality trajectories can be predicted on the basis of known physiological and molecular mechanisms, their interactions with each other, and their interactions with the environment. Hence, the challenge lies in making models as general as possible without loosing sight of what the model is designed to explore. We have aimed to retain generality by making modest assumptions, yet keeping the focus on the envisioned trade-offs.

A model's value is also limited if that what the model aims to explore cannot be found within the model. For instance, a resource allocation model that does not contain enough resources to fulfill some task will unsurprisingly predict that that task is not fulfilled. If the aim of the model was to find out whether that task will be fulfilled or not, the model does not give any additional insight. The sought after limitation was imposed on the model, and an explanation of why the task is not fulfilled cannot be found by studying the model: the model is “inappropriately constrained” [30]. Our model does not include the possibility of negative senescence [19], [30]. Hence, it should be kept in mind that the model does not inform us about the circumstances under which negative senescence could evolve, and that the model in no way excludes this possibility.

When two factors are jointly responsible for affecting fitness, both factors are subject to natural selection [15], [31]. In the model presented here, we imposed *s* and searched for *k* ^*^. Assuming that there is variation in *k*, *k* will tend to evolve in the direction of *k* ^*^. If there is variation in *s*, *s* evolves as well. Organisms with lower *s* will be able to enjoy the benefits of a higher *k* while avoiding some of the costs, and thus enjoy a selective advantage. Although we analyze *k* ^*^ as a function of *s*, we do not chart selection on *s*.

Most studies on adaptive explanations for the evolution of senescence focus on trade-offs between mortality and reproduction [13], [32]. Our study of trade-offs within mortality widens the scope of current modeling of senescence. If a trade-off exists within the mortality function, an increase in fitness derives mostly from lifespan extension. There will be a ‘timing effect’ as well: survival is more evolutionary rewarding if reproduction during the survived ages is high. Yet, notice that the global results that we present here do not depend on the particular pattern of *m*(*x*), but rather on the existence of a trade-off within the mortality function. Our model indicates the global behavior of the model, which depends on the *lifespan extension* achieved if *k* is increased. Thus, we derive the hypothesis that senescence may have evolved because senescent organisms outlive non-senescent organisms. This may be counter-intuitive, but the matter becomes clear when the pace of life is distinguished from the shape of senescence [26]. Pace refers to the amount of time in which a process takes place, for instance the time it takes to live a life. Shape refers to the amount and sort of change that happens during that time, for instance if and how mortality and fecundity change during a lifespan. Lifespan is equal to the inverse of average mortality. If mortality increases over age, but starts off from a much lower level than would otherwise be the case, average mortality may go down, implying lifespan extension.

## Conclusions

1. In the class of trade-offs that we model, the presence as well as the absence of senescence can be predicted by life history optimization, irrespective of function specifics.

2. The highest optimal rate of senescence occurs for trade-offs that entail costs of intermediate severity in terms of senescence.

3. Optimality of senescence depends on the interaction of environment and physiology. Predictions of optimality cannot be derived from either of them alone.

## Supporting Information

Appendix S1 **Formal description of the model.**
(PDF)
