# Supplementary material for: Interaction Mortality: Senescence May Have Evolved because It Increases Lifespan
Source: PLoS One. 2014 Oct 9;9(10):e109638. doi: 10.1371/journal.pone.0109638 (PMC4192302; doi:10.1371/journal.pone.0109638)
Supplement: Appendix S1 — Formal description of the model. (PDF) [file pone.0109638.s001.pdf]

Formal description of the model

There are two parameters, k and s, and one variable, x, that interact in a multiplicative manner: ksx. Parameter k sets the organism up for deterioration over age x, while parameter s indicates the impact of deterioration on mortality. Let

$$y(x; k, s) = ksx. \tag{1}$$

To create the trade-off, mortality is split into two components, A and B. Component $A(y(x; k, s))$ is age-dependent, while $B(k)$ is age-independent:

$$\mu(x; k, s) = A(y(x; k, s)) + B(k). \tag{2}$$

We also assume that mortality is a smooth function.

Parameters k and s are age-independent. The severity of the trade-off is modeled by $s \geq 0$, i.e. s affects the change in component A that results from a given reduction of B by k. Affecting both mortality components, parameter $k \geq 0$ mediates the trade-off:

$$\frac{\partial B}{\partial k} < 0 \ \forall k, \tag{3}$$

and

$$\frac{dA}{dy} > 0 \ \forall y, \tag{4}$$

i.e., if any of x, k and s is increased while the others are non-zero, component A increases.

Mortality cannot be zero or negative. Hence we postulate that

$$B \geq 0 \ \forall k, \tag{5}$$

while

$$A(0) = C > 0. \tag{6}$$

This makes biological sense, because if no deterioration with age occurs ($k = 0$), if deterioration does not lead to age-related increase in mortality ($s = 0$), or if no time has past ($x = 0$), mortality will not (have) change(d), so it will be some constant, C.

Also, the increase in mortality component A should be unbounded:

$$y \to \infty \;\Rightarrow\; A(y) \to \infty. \tag{7}$$

This again makes biological sense, because if the combined effect of the passage of time, deterioration over time, and deterioration affecting mortality goes to infinity, so does mortality.

Finally, let

$$B(0) = E. \tag{8}$$

Thus, the model is defined.

The senescence phenotype is modeled by the parameter settings $[k > 0 \wedge s > 0]$, for if either k or s is zero, $[y = 0\ \forall x] \Rightarrow [A(y) = C\ \forall x]$: $s = 0$ makes mortality insensitive to k (and x) and vica versa.

The physiological constraint s

Lemma 1: $\exists s_z > 0 : k^* > 0$.

Set $r_0 = r_{|k=0}$. For $k = 0$, the effect of a perturbation in k on r is (equation (4) *of the main text*)

$$\frac{\partial r}{\partial k}\Big|_{k=0} = -\frac{\int_0^\infty \left(\int_0^x \frac{\partial \mu}{\partial k}\Big|_{k=0}(t)dt\right) e^{-(r_0+E+C)x} m(x)dx}{\int_0^\infty x e^{-(r_0+E+C)x} m(x)dx}. \tag{9}$$

Element $\frac{\partial \mu}{\partial k}(x)$ of equation (9) can be written as

$$\frac{\partial \mu}{\partial k}(x) = \frac{dB}{dk} + sx\frac{dA}{dy}(y(x)). \tag{10}$$

Note that $y(x; k = 0, s) = 0 \cdot sx = 0$ for all x, s. Hence, with $w_1 = \frac{dB}{dk}|_{k=0} < 0$, $w_2 = \frac{dA}{dy}|_{y=0} > 0$ it holds that

$$\int_0^x \frac{\partial \mu}{\partial k}\Big|_{k=0}(t)dt = w_1 x + s\frac{w_2}{2}x^2. \tag{11}$$

Plugging result (11) back into equation (9) yields

$$\frac{\partial r}{\partial k}\Big|_{k=0} = -w_1 - s\frac{w_2}{2}\frac{\int_0^\infty x^2 e^{-(r_0+E+C)x} m(x)dx}{\int_0^\infty x e^{-(r_0+E+C)x} m(x)dx}. \tag{12}$$

With $-w_1$ strictly positive, it is always possible to pick

$$s_z > 0 : \frac{\partial r}{\partial k}\Big|_{k=0, s=s_z} > 0, \tag{13}$$

implying that

$$\exists s_z > 0 : k^* > 0, \tag{14}$$

which is what we set out to prove.

Lemma 2: $\exists s_M > 0 : \forall s \geq s_M : k^* = 0$.

Suppose $\tilde{\mu} = A$ (mortality supposing there were no B). Then $\tilde{\mu}$ gives $\tilde{r}(k, s)$. Since $\mu(x) \geq \tilde{\mu}(x)$ and $l(x) \leq \tilde{l}(x)\ \forall x > 0$, it holds that

$$r(k, s) \leq \tilde{r}(k, s)\ \forall k, s. \tag{15}$$

Given $s > 0$, any increase in k increases $\tilde{\mu}$ at all ages except age 0, which implies

$$\forall s > 0\, \forall k : \frac{\partial \tilde{r}}{\partial k} < 0\ \forall k\ . \tag{16}$$

Given $k > 0$, any increase in s increases μ and $\tilde{\mu}$ at all ages except age 0, so that

$$\forall k > 0\, \forall s : \frac{\partial r}{\partial s} < 0 \text{ and } \frac{\partial \tilde{r}}{\partial s} < 0. \tag{17}$$

Condition (7) implies that

$$\forall k > 0 : \lim_{s \to \infty} \tilde{r}(k, s) = -\infty. \tag{18}$$

Given equation (12), one can choose s big enough so that $\frac{\partial r}{\partial k}|_{k=0}$ is negative:

$$\exists s_1 : \frac{\partial r}{\partial k}\Big|_{k=0} < 0. \tag{19}$$

Together with (17), this implies

$$\exists k_1\ \forall s \geq s_1 : \forall 0 < k \leq k_1 : r(k, s) \leq r(k, s_1) < r_0. \tag{20}$$

Properties (17) and (18) imply that

$$\exists s_2 : \forall s \geq s_2 : \tilde{r}(k_1, s) \leq \tilde{r}(k_1, s_2) < r_0. \tag{21}$$

Now choose

$$s_M = \max\{s_1, s_2\}. \tag{22}$$

Then $\forall s > s_M$:

$$(i) \quad 0 < k \leq k_1 : r(k,s) < r_0 \text{ (because of (20))} \tag{23}$$

$$(ii) \quad k > k_1 : r(k,s) < \tilde{r}(k,s) \leq \tilde{r}(k_1,s) < r_0 \text{ (because of (21))}, \tag{24}$$

implying that

$$[\forall s \geq s_M : \forall k > 0 : r(k,s) < r_0] \Rightarrow k^*(s) = 0. \tag{25}$$

This completes the proof.

The environment

How would variation in the environment affect the results? Referring to equations (9) and (10), the general answer is that this depends on how dB/dk depends on the environment. For instance, let

$$B(k,E) = E/(k+1), \tag{26}$$

so that

$$\frac{\partial}{\partial E}\left(\frac{\partial B}{\partial k}\right) < 0, \tag{27}$$

$$E \to \infty \Rightarrow \frac{\partial B}{\partial k} \to -\infty. \tag{28}$$

This implies (equation (12)) that for every specified $s > 0$

$$\left[\exists E : \left.\frac{\partial r}{\partial k}\right|_{k=0} = -w_1 - s\frac{w_2}{2}\frac{\int_0^\infty x^2 e^{-(r_0+E+C)x} m(x)dx}{\int_0^\infty x e^{-(r_0+E+C)x} m(x)dx} > 0\right] \Rightarrow k^* s > 0, \tag{29}$$

so that the environment can always be harsh enough to lead to senescence. If, on the other hand,

$$B(k, E) = E - k, \tag{30}$$

with $k < E$, it holds that

$$\forall E \, \forall k : dB/dk = -1, \tag{31}$$

in which case E does not affect k^*.
